# Adropin and Irisin in Patients with Cardiac Cachexia

**DOI:** 10.5935/abc.20180121

**Published:** 2018-07

**Authors:** Alfredo José Mansur

**Affiliations:** Instituto do Coração (InCor) do Hospital das Clínicas da Faculdade de Medicina da Universidade de São Paulo (HC FMUSP), São Paulo, SP – Brazil

**Keywords:** Heart Failure, Adropine, Cachexia, Prognosis, Weight Loss

Cardiological clinical practice involves the care of patients with heart failure who lose weight, which not rarely culminates in cardiac cachexia. The differential diagnosis with other consumptive disorders can lead to an extensive diagnostic investigation.

**Figure 1:**
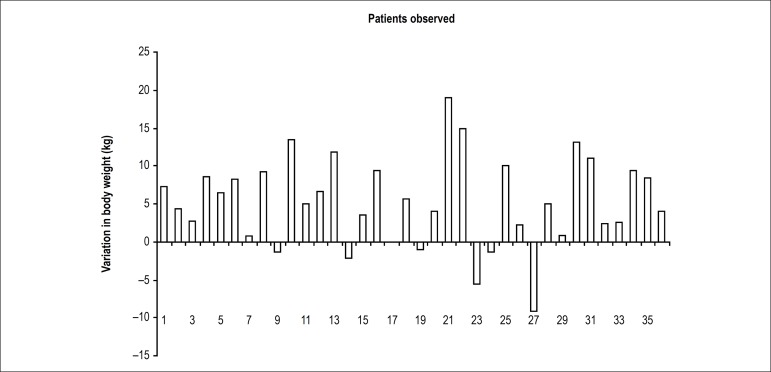
Body weight variation in 2 observations.

That has been a theme of interest in the medical literature for decades,^1^ and its importance remains recognized over time.^2-7^ Physicians with decades of experience in cardiological clinical practice have noticed that individuals with heart failure due to heart valvular disease gain weight after well-succeeded surgical interventions that reverse heart failure. In other words, heart failure reversion also manifests as weight gain. A recent outpatient clinical observation [Correia GF & Lima NNC, unpublished data] of 36 patients for months has found body weight variation with the current pharmacological treatment for heart failure including betablockers (Figure 1).

Different metabolic mechanisms can mediate that clinical manifestation.^8,9^

In this issue of the *Arquivos Brasileiros de Cardiologia*, Kalkan et al.^10^ have added to the studies in the area the results of the research on two proteins that act on the mechanisms of energetic homeostasis – adropin^11^ and irisin.^12^ Those authors have found that the concentration of those proteins differed in 44 patients with cachexia (body mass index 19.9; standard deviation 1.12) as compared to that of 42 patients without cachexia (body mass index 29.2; standard deviation 4.25). On multivariate logistic regression, adropin remained associated with cachexia, despite the low hazard ratio.

Some limitations of the study by Kalkan et al.^10^ were the lack of information about the etiology of heart failure, the small sample size and the lack of log-term follow-up data. Therefore, the results presented, although initial and exploratory, are important, and further studies should be conducted to elucidate the metabolic mechanisms of weight loss in patients with heart failure.
